# A Boundary Tool for Multi-stakeholder Sustainable Business Model Innovation

**DOI:** 10.1007/s43615-021-00103-3

**Published:** 2021-09-14

**Authors:** M. G. E. Velter, V. Bitzer, N. M. P. Bocken

**Affiliations:** 1 Maastricht Sustainability Institute, Maastricht University, P.O. Box 616, 6200 MD Maastricht, The Netherlands; 2 Fontys Centre of Expertise on Circular Transitions, Fontys University of Applied Sciences, De Lismortel 25, 5612 AR Eindhoven, The Netherlands; 3 KIT Royal Tropical Institute, Mauritskade 64, 1092 AD Amsterdam, The Netherlands; 4 International Institute for Industrial Environmental Economics (IIIEE), Lund University, Box 196 221 00, Lund, Sweden

**Keywords:** Process tool, Boundary work, Sustainable business model innovation, Circular business model innovation, Multi-stakeholder collaboration

## Abstract

Sustainable business model innovation cannot reach its full sustainability potential if it neglects the importance of multi-stakeholder alignment. Several studies emphasize the need for multi-stakeholder collaboration to enable sustainable business model innovation, but few studies offer guidance to companies for engaging in such a collaborative process. Based on the concept of boundary work, this study presents a tested process tool that helps companies engage with multiple stakeholders to innovate sustainable business models. The tool was developed in three iterative phases, including testing and evaluation with 74 participants in six sustainable business model innovation cases. The final process tool consists of five steps to facilitate multi-stakeholder alignment for sustainable business model innovation: (1) defining a collective ambition, (2) mapping and negotiating the changing organizational boundaries, (3) exploring opportunities and tensions for aligning stakeholders, (4) defining first interventions and (5) developing a collaboration pitch. We found that the tool enables discussions and negotiations on sensitive topics, such as power reconfigurations and mutual responsibilities to help stakeholders align. For companies, the boundary tool enriches sustainable business model innovation by offering guidance in the process of redesigning their multi-stakeholder system, assessing their own organizational boundaries, exploring, negotiating and prioritizing strategic actions based on organizational boundary changes and kick-starting new partnerships.

## Introduction

Over the past few years, sustainable business model innovation (SBMI) has gained attention as a necessary means to contribute to sustainable development and the circular economy [1–4]. To reach this sustainability potential, multiple studies have found that this requires a collaborative process which innovates the business model of the initiating company [5–7] and aligns the business models of multiple stakeholders [8–10]. However, such a multi-stakeholder collaborative process is extremely difficult in practice as stakeholders often have different priorities, value logics and business models that pose significant challenges in finding mutual value opportunities and overcoming barriers for alignment [9, 11]. As a result, many companies tend to set the scope of their SBMI process rather narrowly to avoid having to deal with multiple stakeholders in lengthy and complicated processes [12]. Instead, they often opt for bilateral collaborations with producers and customers, which leads to incremental innovations rather than exploring more far-reaching innovations necessary for sustainable business models [12–14]. There is thus a crucial need to broaden the scope of SBMI and support companies in collaborating with critical stakeholders [13, 15, 16]. While important work is being done to assist individual organizations involved in processes of SBMI [17–19], a multi-stakeholder approach that supports the development of collaborative stakeholder environments for this purpose is missing [2, 20–22].

To address this gap, the goal of this paper is to present a practical tool designed to support companies (or other organizations) in sustainable business model innovation based on a multi-stakeholder approach. The tool draws on boundary work, as a suitable perspective that supports exploring, negotiating and implementing multi-stakeholder interactions on organizational boundaries [23–25]. We focus on the changing organizational boundaries of *identity* (the choice of ‘who we are’), *power* (the ability to control relationships), *competence* (resources, capabilities, knowledge) and *efficiency* (activities and transactions). These types of boundaries have been identified as critical elements in processes of sustainable business model innovation [23, 25].

The boundary tool presented in this paper addresses the changing and interdependent stakeholder boundaries involved in collaborative SBMI. This helps companies to engage with multiple stakeholders and improve alignment for the purpose of SBMI. The tool has been thoroughly tested in a design science research process with 74 participants from multiple organizations of different sizes, from different sectors and with different organizational characters (companies, intermediaries, NGOs, educational and research institutes, etc.). Testing comprised six sustainable business model innovation cases with companies that needed, or were involved in, boundary work with multiple stakeholders. While aiming at innovation managers in companies, the tool may also inspire innovation with NGOs, citizen initiatives and policy makers.

To develop the boundary tool, this article first describes the need for a tool for multi-stakeholder SBMI and the potential of the boundary work framework to address this gap, which leads to the objectives of the tool. Subsequently, the research method describes the approach of this study and its processes of data collection. This is followed by a description of the findings and implications for tool development in different stages of the research. The article then discusses the contributions of the paper to the theoretical and practical domain of SBMI, the implications and limitations of the study and avenues for further research. The article ends with the conclusion and the key benefits of the boundary tool for companies.

## Theoretical Framework

### The Need for a Tool for Multi-stakeholder SBMI

The redesign of a company’s stakeholder network is a key challenge for SBMI [21, 26, 27]. Due to this complexity, there are different SBMI tools that take into account the company’s external stakeholders in an implicit or explicit manner (Table 1).

**Table 1:** Review of SBMI tools. Developed from [2, 19, 20, 28–30]

**Tool name**	**Author(s)**	**Type of tool**	**Tool objective**	**Perspective on external stakeholders**	**Stakeholder focus**	**Stakeholder inclusion**
BM3C2 framework	[30]	Strategy tool	Visualizing the parties involved, connecting and aligning their business models	Value flows, resources and competences, internal organization	Explicit	Multi-stakeholder focus possible
Circular innovation ecosystem tool	[31]	Creative tool	Analyze, ideate and develop the circularity potential of the innovation ecosystem	Circular strategies	Implicit	Depending on participant ideas
Value mapping tool	[32]	Creative and Assessment tool	Understand positive and negative aspects of the value proposition, identify conflicting values and opportunities for business model redesign	Value created, missed, wasted, destroyed and new value opportunities	Explicit	Multi-stakeholder focus
Triple-layered business model canvas	[33]	Creative tool	Support the creative exploration of sustainable business models and sustainability-oriented innovation	Value created	Explicit for particular actors	Limited to particular stakeholders
Ecosystem pie model	[34]	Strategy tool	Map, analyze and design innovation ecosystems	Resources, activities, value addition, value capture	Explicit	Variably
Circular collaboration canvas	[17]	Creative tool	Think in detail upon a circular value proposition	Power, value proposition	Implicit, compounded	No (multi-) stakeholder focus
Collaboration tool for the building sector	[18]	Process tool	Enhance collaboration for CE in the building sector	Visions, activities	No actor focus	Limited to market & supply chain
Circular business model mapping tool	[35]	Creative tool	Offer a standardized representation of elements and possible cycles of circular business models	Value proposition, value creation & delivery, value capture	Implicit, compounded	No (multi-) stakeholder focus
Flourishing business canvas	[33]	Creative tool	Modeling across living ecosystems and social systems	Value co-creations, value co-destructions	Implicit, compounded	Stakeholders and ecosystem actors
Sustainability innovation pack	[36]	Assessment tool	Review shared values for modeling SBMs	Touchpoints	Implicit, compounded	No (multi-) stakeholder focus
Sustainable business canvas	[37, 38]	Creative tool	Support entrepreneurs and start-ups in their design of SBMs	Value propositions, power	Implicit, compounded	No (multi-) stakeholder focus
BMC infrastructure	[39]	Creative tool	Designing infrastructure business models that incorporate economic, social and environmental aspects	Value streams and propositions	Implicit, compounded	No (multi-) stakeholder focus
SBM pilot canvas	[40]	Process tool	Translate sustainable business model ideas into small-scale pilots	Resources, actions	Implicit, compounded	No (multi-) stakeholder focus
Degree of engagement diagram	[19]	Strategy and process tool	Enable stakeholder engagement degrees	Roles	Explicit	Multi-stakeholder focus

Tools are artefacts that can support understanding, assessment, creativity and/or change on particular practices, such as (broadly applicable) guidelines, checklists or artefacts with an analytical focus [20, 41]. However, existing tools for SBMI generally address the value creation & delivery system in an implicit way and with a focus on value innovation. Fewer tools are available that illuminate and address the interdependencies between stakeholders [19, 30, 32, 34]. For example, the value mapping tool [32] explores the multi-stakeholder network on value propositions, the ecosystem pie includes external stakeholders’ resources and activities that can be utilized to create and capture value [34] and the degree of engagement diagram [19] guides practitioners with engaging multiple stakeholders along the SBMI process. The sustainable business model tool elicits tangible resources and competences as well as internal costs and impacts in relation to a common objective [30]. While these tools are suited for innovating the multi-stakeholder value aspects of SBMI, they overlook interdependent and underlying issues of power, identity and competences of the various stakeholders that influence the alignment of these stakeholders [9, 42]. This study addresses this gap by developing a practical tool that assists companies in (re-)aligning their own organizational boundaries in coherence with reconfigurations in their multi-stakeholder network. The tool is developed based on the boundary work approach that integrates boundary phenomena in contemporary multi-stakeholder environments [24, 25].

### A Boundary Work Approach to Sustainable Business Model Innovation

SBMI necessitates multi-stakeholder collaboration to realign organizational boundaries, yet prevailing studies fall short with tools that help practitioners to engage in such boundary work [2, 20–22]. Boundary work is a highly iterative process of navigating mutually dependent values, strategies and concrete actions of interdependent stakeholders without external control [43]. Recent studies on SBMI describe boundary work as the iterative activities of e*xploring*, *negotiating*, *disrupting* and *re-aligning* organizational boundaries around sustainable value propositions and capture mechanisms [25]. To develop a tool that assists such reconfiguration processes for SBMI, this study sources from organizational boundary theory to define *architectural* building blocks, and from boundary work theory to define *processual* building blocks.

**Fig. 1:**
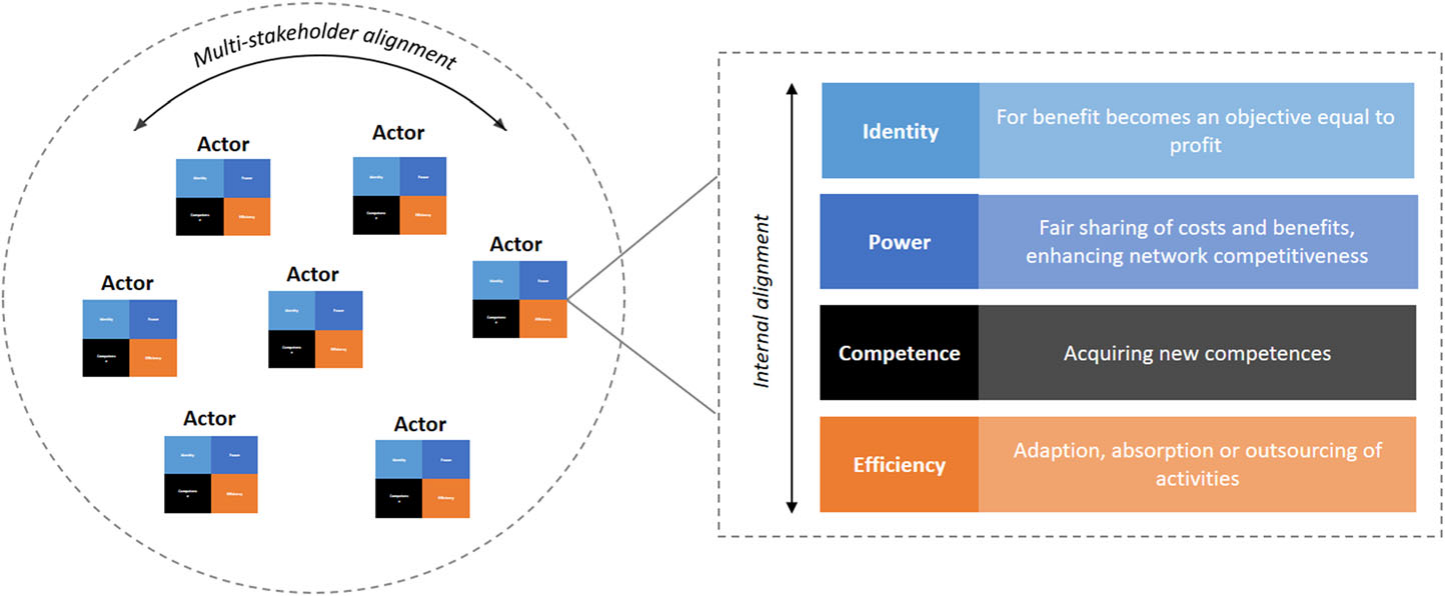
Framework for organizational boundary alignment in SBMI. Based on [23–25]

On the architectural side, SBMI affects organizational boundaries of identity, power, competence and efficiency [23–25]. On the identity dimension, typical boundary reconfigurations are the integration of a social and environmental objective in the normative orientations of the organization (e.g. mission, vision, value statements), and which is shared between organizations to develop stakeholder networks [28, 44]. The power boundary is typically reconfigured by a focus on network competitiveness and long-term contracts with a large element of trust instead of individual power accumulation and transactional relationships [45]. The boundary of competence typically shifts towards inclusion of repair and remanufacture skills, circular design, modular processing, but also more intangible aspects such as network collaboration, dynamic capabilities [46, 47] and experimentation capabilities [44, 48, 49]. On the efficiency boundary, SBMI promotes a shift in the division of roles and activities. While each boundary dimension deals with a different organizational issue (i.e. coherence on identity, autonomy for power, growth for competence and transaction costs for efficiency), they are interdependent within the organization and between organizations (Figure 1).

SBMI thus not only requires changes in the boundaries of the initiating organization, but develops in conjunction with boundary changes of network actors, such as other companies, civil society organizations, policy makers and intermediaries [24, 50]. It is likely to encounter clashes with current organizational boundary configurations and synergies on others [51]. A boundary work process can help companies in such reconfiguration processes through processes of organizational boundary exploration, negotiation and reconfiguration around an SBMI ambition. Organizational boundary exploration helps to make boundary-spanning interactions and relations explicit, which assists the identification of the potential partner contributions, the different tensions to be addressed and shifts individual problems to collective problems to be dealt with [21]. The latter is particularly relevant for innovation processes with a wide variety of stakeholders and that aim to exceed incremental forms of innovation [52, 53]. The negotiation of organizational boundary changes aims to reconcile tensions between current and envisioned organizational boundaries and to define interventions that accommodate these tensions. The reconfiguration of organizational boundaries happens through agreement, experimentation and embedding of the changes [26, 54].

By bringing together the architectural and processual elements, we regard SBMI as a multi-actor boundary work process that requires extensive networking and continuous exploration and negotiation of mutual boundary reconfigurations in parallel to organizational boundary shifts that contribute to the development and implementation of sustainable business models. The notion of boundary work highlights that current boundary configurations and their interfaces can function as a lock-in for SBMI, whereas actively working on organizational boundaries could potentially function as a source of innovation.

### Research Gap: a Tool for Multi-stakeholder Sustainable Business Model Innovation

SBMI not only requires changes within a single organization, but also develops in conjunction with changes in the stakeholder network. These changes can be processes or activities (for example, producers experiment with recycled materials), skills (for example, recyclers need different processing methods), roles (for example, the role of system orchestrater, or service provider in addition to an existing role) and sometimes even a modified identity, by integrating a social purpose in the mission and vision of the organization. These changes all have to do with changes in organizational boundaries. The creation of a sustainable business model therefore requires a process of exploring, negotiating and implementing organizational changes. As part of this process, this study presents two gaps on SBMI:

1. The theoretical gap of a lack of approaches that integrate boundary phenomena of stakeholders in contemporary multi-stakeholder environments.
2. The practical gap of companies lacking tools to engage with their multi-stakeholder network to innovate sustainable business models.

The purpose of this study is to address these gaps by developing a practical tool that assists companies in realigning their own organizational boundaries in coherence with reconfigurations in their multi-stakeholder network, to innovate sustainable business models. The potential of boundary work for SBMI to contribute to these gaps lies in its two-stream approach of, first, the boundary work *process*, being the exploration and negotiation of actor boundaries to promote boundary alignment, and second, the inclusion of *architectural* changes in organizational boundaries of the initiating firm and its stakeholders to develop sustainable business model(s).

The purpose of the tool is to assist companies in aligning multiple stakeholders for the innovation of sustainable business models. It aims to do so by integrating the changing and interdependent organizational boundary phenomena in SBMI on the company and multi-stakeholder level. As part of this process, this tool focuses on:

1. crafting a collective SBMI ambition
2. mapping and negotiating the changing organizational boundaries of the individual organization and its multi-stakeholder network
3. exploring opportunities and tensions for stakeholder alignment
4. defining first interventions
5. forming a collaboration pitch

We used the checklist for sustainability tool development [20] to determine the practical requirements of the tool: the tool should be easy to use, adaptable to the different SBMI ideas and stakeholder contexts of the users and feasible to apply within a single workshop timeframe of max. 3 h.

## Methods

### Research Approach

This study used design science research (DSR) to develop the boundary work tool [42, 55]. DSR intends to create artefacts as objects that embed solutions to an understood research problem [56]. According to DSR, the artefact development should be a search process drawing from existing theory and knowledge to come up with a solution to a defined problem, with its utility, quality and efficacy thoroughly evaluated. DSR facilitates a thorough scientific and empirical process of developing such an artefact, consisting of six steps of problem identification and motivation, definition of objectives for a solution, design and development, demonstration, evaluation and communication [57].

The ability of DSR to integrate science and practice in developing solutions for stakeholders and users makes it appropriate for the development of a boundary work tool [42, 56]. In addition, in the context of boundary work for SBMI, such artefacts can function as boundary objects for societal problems too, on which the multiple stakeholders involved can frame, shape, refer to and use as means to accommodate organizational boundary changes [58–62].

### Tool Development Process

**Fig. 2:**
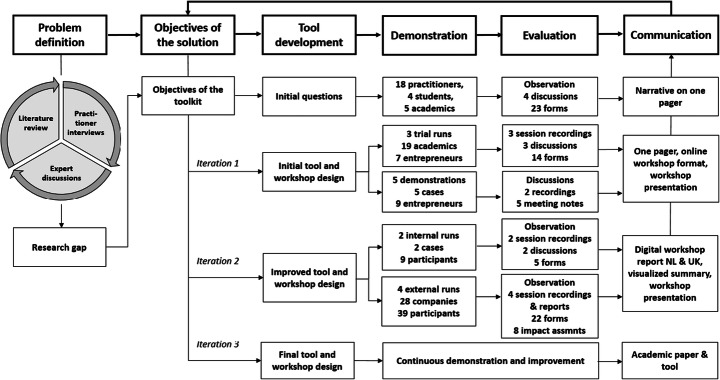
Tool development process applied in this study

We followed the stepwise DSR approach to develop the tool (Figure 2). In total, nine workshops were held involving 74 participants from six SBMI cases, where a case constitutes an initiating company and its various stakeholders. The workshops were facilitated by the first author. All workshops took place in the first quarter of 2021 and took between 1.5 and 2.5 h. Due to the COVID-19 crisis, all sessions were held digitally.

#### Start of the Process

We started the process by studying the practical problem of aligning external stakeholders for SBMI through a literature review, multiple practitioner interviews and discussions on topics of the framework [25, 63]. This led to the development of first objectives of the tool. Based on these objectives, we organized four expert panel groups with a total of 28 participants: eight entrepreneurs, three innovation managers, five sustainability managers, three intermediaries, four design students and five researchers in the field of Sustainability Transitions and Sustainability-oriented Innovation. Most of the participants did not know each other beforehand and were not familiar with each other’s SBMI trajectories. The expert panels comprised 2-h meetings of exploratory discussions on central elements of the framework. In addition to companies, each panel involved at least two other types of actors, such as an NGO, intermediary actor, academics and students. We used the software Mentimeter to pose questions centered on elements of the boundary work framework. Practitioners shared their experiences and needs regarding stakeholder alignment and sustainable business model innovation taking the Mentimeter questions as the starting point. This evaluation helped to refine the objectives of the solution and understand the different contexts and linguistic frames used by the participants, leading to an initial tool and workshop design.

#### First Iteration of the Tool

We tested the initial tool and setup of the workshop in three trial runs with 16 participants in total. Two trial runs were held with academics and PhD students of sustainability-related research fields. As part of the Dutch Circular Economy Week, entrepreneurs, students, consultants, intermediaries and teachers joined the third trial run. Each trial run had a different fictional SBMI topic and stakeholder network. We evaluated the workshop using recordings, discussions at the end of the workshop and a questionnaire that inquired the relevance of the tool for the targeted purpose, ease of use, conceptual clarity, learnings and recommendations (Appendix 1). In addition to the trial runs, we demonstrated and discussed the workshop setup and tool with five companies, where we placed the tool in their SBMI context and gathered insight on the relevance of the tool for their organization, and improvements in setup and design of the tool. Evaluation took place using the discussions, recordings and meeting notes and led to an improved tool and workshop design.

#### Second Iteration of the Tool

We tested the improved tool in six Dutch SBMI cases: in two internal workshops at companies, and four workshops with companies and their external stakeholders. The contexts in which the workshops were applied are as follows:

- *Arapaha:* A purpose-driven start-up that develops zero-waste, climate friendly and fossil-free consumer products wanted to explore their own boundary setting in relation to their network and identify stakeholder tensions to enhance circularity along the design, production and consumption of their products. Internal workshop consisting of the two directors and a communications employee (three participants).
- *Lightronics:* A medium-sized lighting solution company that wanted to explore how to develop business models for future-proof and circular street lighting that takes into account interests of the users, surrounding residents and the natural environment. Internal workshop consisting of a sustainability manager, smart solutions consultant, tender manager and three circular economy students (six participants).
- *Lightronics:* For the same company, the internal workshop was followed up by an external workshop with the initiating company, a light pole company and an educational institute (eight participants). The workshop focused on organizing the return process for street luminaires.
- *Arveco:* A medium-sized company-clothing distributor that wanted to develop a circular business model and used the workshop to explore how they together with network partners can collaborate to use company clothing for as long as possible. External workshop with the clothing distributor, reversed logistics provider, potential customers, funding company, intermediary, clothing producer, innovation company, educational institute (11 participants).
- *Philips:* A large health technology company that wanted to develop a structural way of reusing healthcare equipment in underserved communities. External workshop with a refurbished systems manager, access to care manager, medical donation NGOs, academic hospital, recycling company, innovation company, research institute and visual illustrator (11 participants).
- *City Farm Brandevoort:* A citizen initiative that aimed to develop a business model for a city farm. They were looking for partners to join this initiative and contribute to the social, sustainability and health ambitions of the city farm. External workshop with the initiators, an educational institute, horeca entrepreneur, coffee roaster, social workplace, green maintenance company and visual illustrator (9 participants).

We used observations, session recordings, digital reports of the content of the sessions and an extended questionnaire (Appendix 2) to evaluate and adjust the workshop and tool. In addition, we inquired with the initiators about the impact on personal realizations, follow-up meetings and choices made after the workshop.

#### Third Iteration and Final Tool Development

The previous iterations led to the final tool and workshop design and accompanying files presented in this paper. The tool and workshop can be continuously demonstrated and improved.

## Results

### The Boundary Tool

**Fig. 3:**
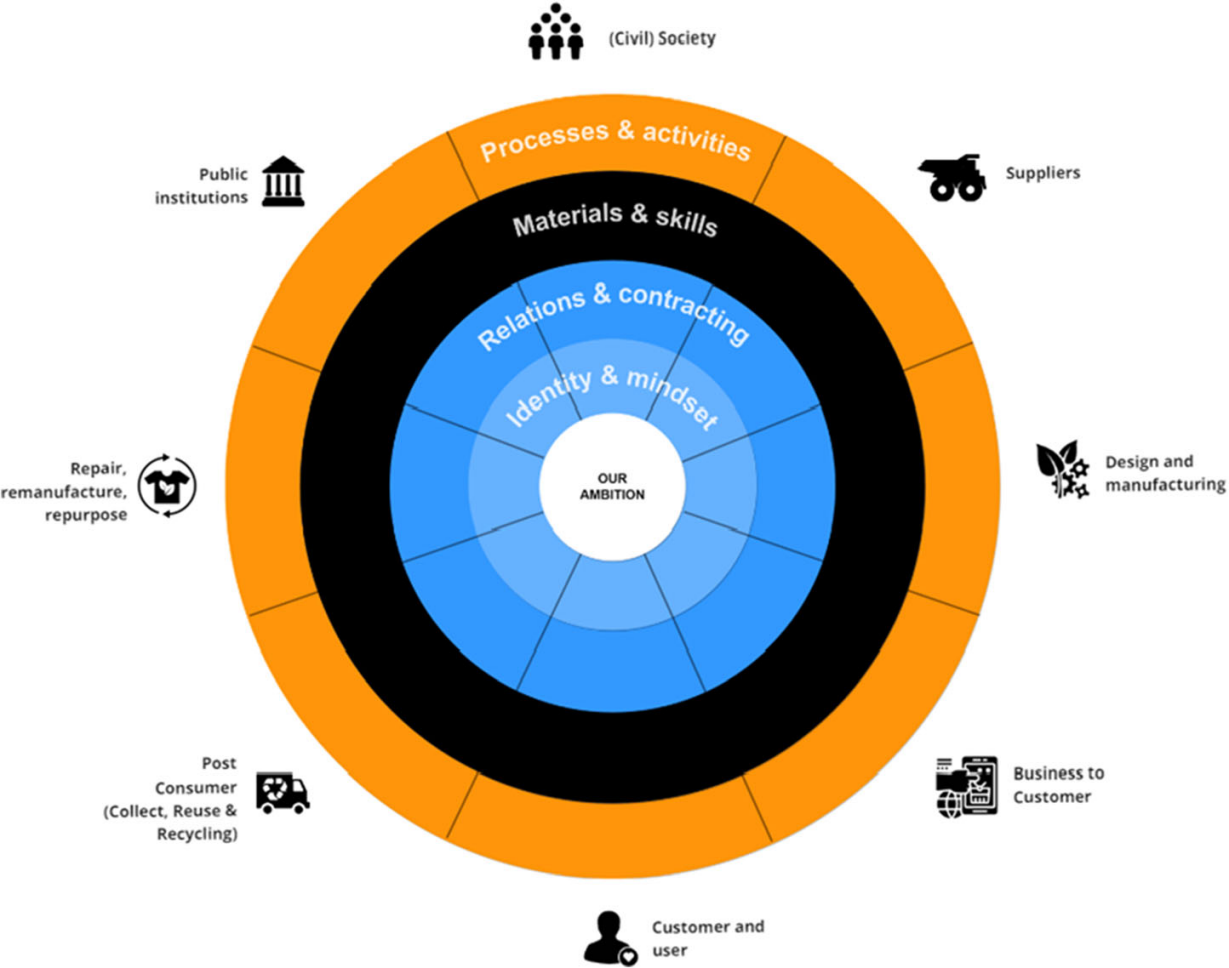
Boundary tool

**Fig. 4:**
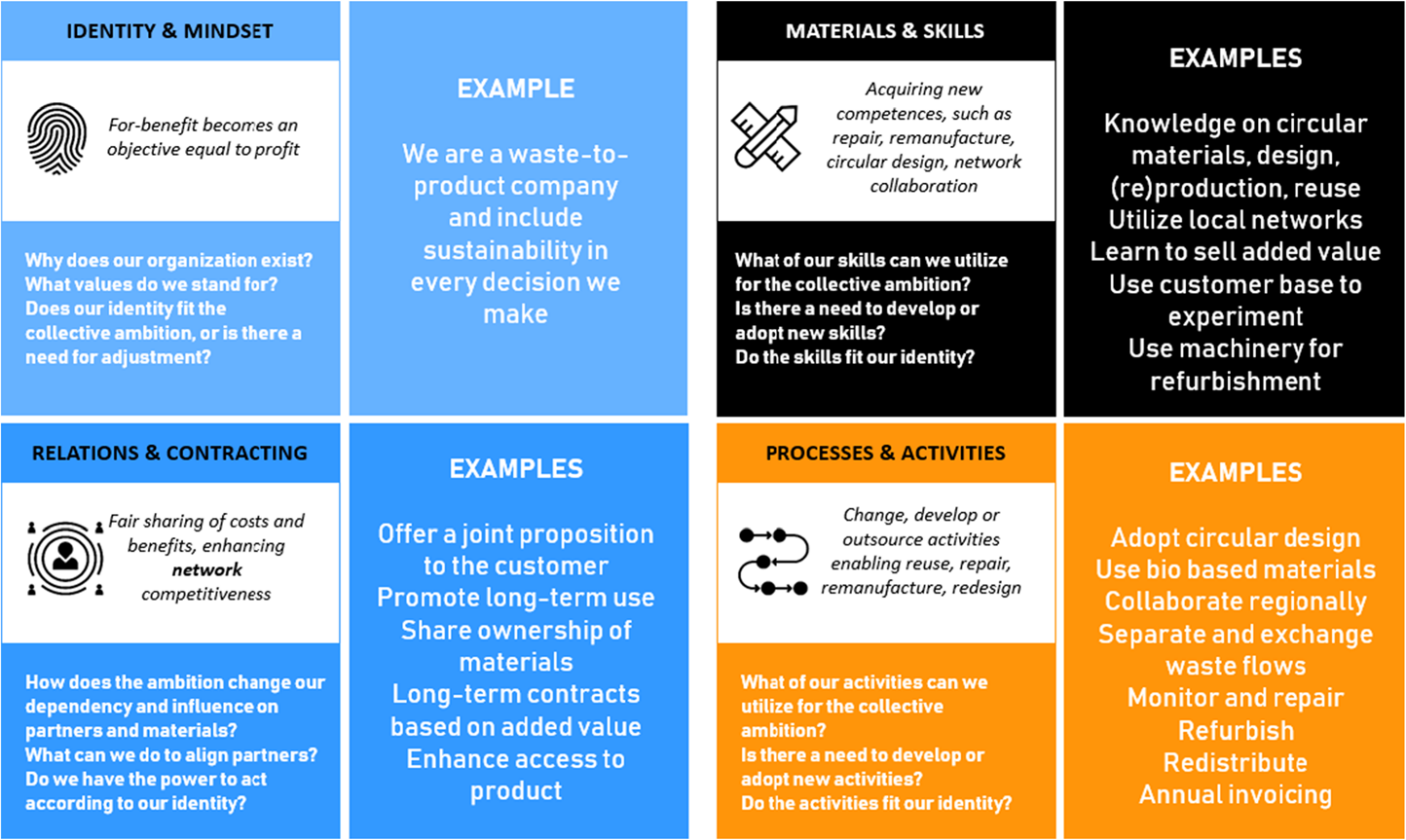
Organizational boundary cards

The boundary tool is conceived to help companies engaging in or intensifying multi-stakeholder collaborations for sustainable business model innovation. The tool features a circle that centers on a collective ambition and is surrounded by four different organizational boundary dimensions (Figure 3). The boundary dimensions have a distinct color: light blue represents ‘identity and mindset’, dark blue represents ‘power’, black represents ‘competence’ and orange represents ‘efficiency’. The names of the boundaries’ dimensions have been adjusted based on the linguistic frames of the participants. A populated example of the tool can be found in Appendix 3. Organizational boundary cards with similar colors support the tool (Figure 4). Each card contains the title of the boundary, a description of the typical reconfiguration and guiding questions that help participants to map the focal boundary changes and reflect on its internal alignment. The backside of the cards contains one or more examples of boundary changes.

### The Process of Using the Tool

The boundary work tool can be used in a workshop with multiple type of stakeholders, such as companies, NGOs, governmental agencies, consumers, and research and educational institutes. These stakeholders do not have to be collaborating with the initiating company yet and can even be stakeholders from other sectors that have the potential to be relevant for the SBMI case, such as remanufacturing or recycling organizations. The workshop setting is chosen to facilitate debate and collaborative discussions on issues that a single stakeholder might face.

#### Pre-workshop Preparation

In preparation of the workshop, a discussion should take place with the initiating company to frame the problem and manage expectations of the workshop. The problem can be framed by discussing the SBMI ambition, the relevant stakeholders (collaborators and non-collaborators) and the main issues that the company faces in aligning these stakeholders. In these conversations, it is important to include a multi-stakeholder perspective by explicitly asking for the current and expected role of these stakeholders. Based on this conversation, potential participants are identified, placed in the tool and invited for the workshop. The expectations can be managed by presenting the boundary framework and workshop setup to the initiators, and asking them when they perceive the workshop successful. We also sent a one-pager containing the workshop description and relevance to the participants beforehand.

#### Workshop Process

Applying the tool consists of seven steps:

1. Setting the scene and getting started.
  The goal of this step is to introduce the topic of the workshop, to familiarize the participants with the boundary concepts and the different steps of the workshop, and to introduce the participants to each other. Depending on whether stakeholders already know each other, the role of the facilitator adjusts. For example, when the stakeholders are unfamiliar with each other, the facilitator functions as an intermediary that connects and involves the different stakeholders.
2. Developing a collective SBMI ambition.
  The goal of this step is to formulate the SBMI ambition of the multi-stakeholder network, which guides the development of opportunities for collaboration in the next steps. We challenge the participants to include both profit, social and environmental aspects, and to think about their potential modest and advanced improvements that contribute to the ambition.
3. Exploring the changing organizational boundaries.
  The goal of this exercise is to explore how the ambition affects the organizational boundaries of the individual organizations in the multi-stakeholder network. Participants can use the organizational boundary cards to map their existing boundaries, but probably the proposed SBMI ambition requires them to adjust their existing boundaries. The mapping is followed up by a discussion on the boundary changes.
4. Negotiating boundary changes and identifying matches and mismatches.
  This step aims to negotiate the required boundary changes and identify opportunities (matches) and tensions (mismatches) between stakeholders. The discussion about boundary changes often intrinsically leads to the identification of matches and mismatches, which can be written down on separate post-its. Be aware that matches and mismatches can relate to multiple instead of bilateral boundary changes or to a sequence of boundary changes to be made, but try to specify the ‘we will if you all will’ notion to actors as far as possible. This may be repeated several times throughout the collaboration to enhance collective action [64].
5. Prioritizing matches and mismatches.
  The goal of this step is to make a selection of the matches and mismatches to continue with. Participants can use voting dots to cast their vote on matches and mismatches that they think are most important to take further. In our experience, an amount of three matches/mismatches to define interventions on provides a solid base to proceed with.
6. Defining first interventions.
  The goal of this step is to define first interventions on the matches/mismatches that help to implement the novel organizational boundaries. In addition to steps the participants themselves can take, this can also lead to the need to involve additional stakeholders, to acquire additional knowledge or to revise the ambition.
7. Drafting a collaboration pitch. The goal of this step is to provide the participants with a clear starting point for collaboration. Using the collaboration pitch format (Appendix 4), participants summarize the workshop by defining a collaboration pitch that includes the collective ambition, specific interventions and the actions that the relevant stakeholders aim to take to proceed the collaboration.

The stepwise description of the workshop setup for a 2.5-h setting is provided in Table 2. The workshop should be guided by a skilled facilitator who is familiar with the organizational boundary framework. To assist facilitation, we provided detailed lines and probing questions for each of the steps in Appendix 5.

**Table 2:**
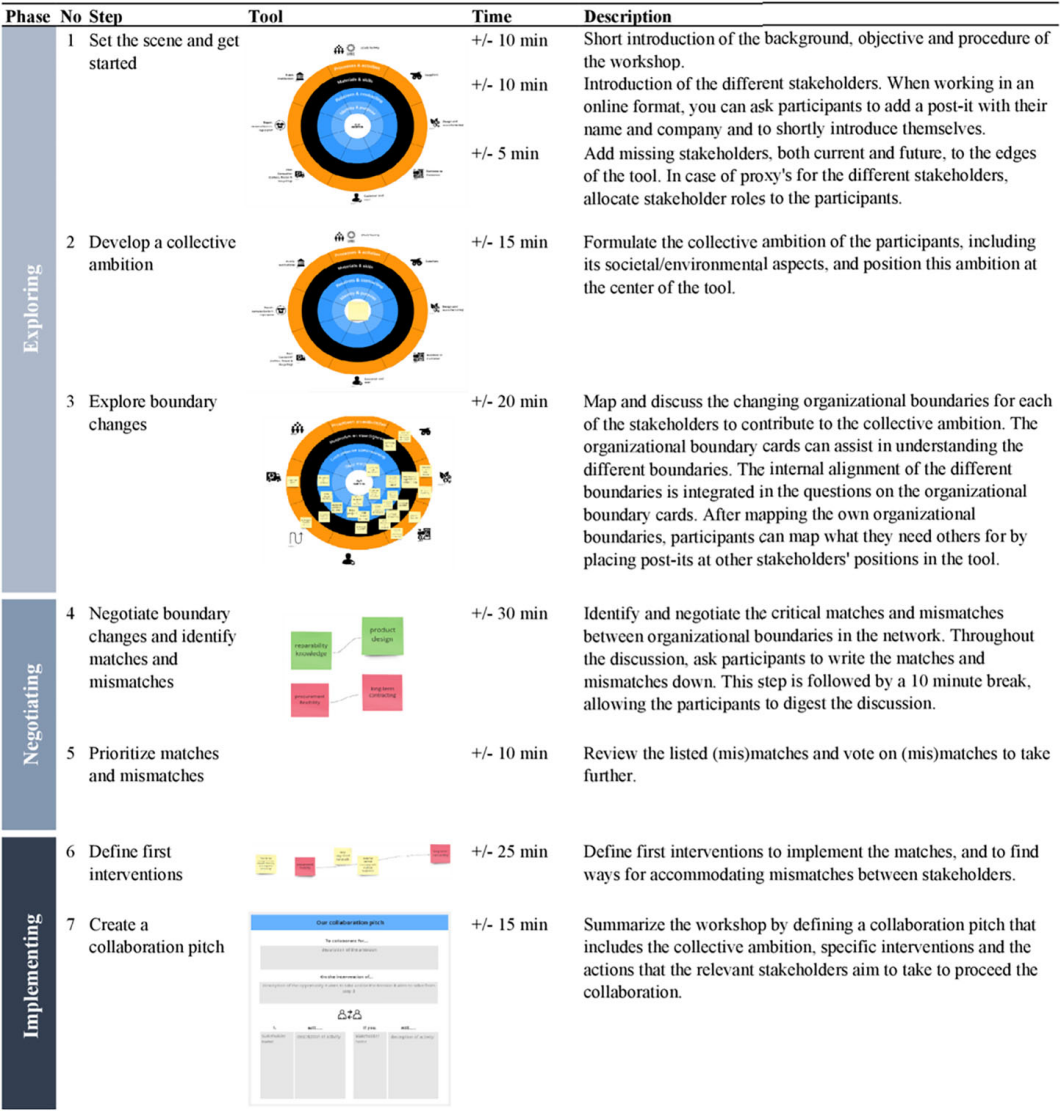
A detailed description of the different workshop steps in a typical tested format

### Tool Evaluations and Adjustments

After each iteration in the tool development process, the tool has been evaluated using observations, discussions and questionnaires. Ultimately, we arrive at an average evaluation of the final tool of 2.1 (*σ*= 1.04) on a scale from 1 to 5, where ‘1’ means ‘fully agree’ and ‘5’ means ‘fully disagree’, where the support of the boundary cards and the ease of use scored best (Table 3). Participants of the SBMI cases appreciated the focus on organizational boundaries, expressing that the tool provided *“deeper insights in the potential to leverage other organizations’ strengths and opportunities*” (participant 1 of Philips workshop, 15-04-2021), that it was *“excellent to work down from identity & purpose. And to understand common ground”* (participant 2 of Philips workshop, 15-04-2021), that the tool helped to *“find opportunities and difficulties in the boundaries”* (participant 3 of Philips workshop, 15-04-2021) and to *“Make a clearer distinction between certain ideas and where they belong in the organization”* (participant of Lightronics workshop, 24-02-2021)*.* Participants also appreciated the tool’s ability to connect to internal and external stakeholders, something that would otherwise be challenging to organize, as illustrated by a participant of the CityFarm Brandevoort workshop: *“How beautiful it is to work together across borders. By exploring this in this way you can get to know each other and our cooperation opportunities”* (30-03-2021) and by a participant of the Arveco workshop: *“The tool shows how we can tackle obstacles together. Through consultation with various parties it suddenly becomes clear where opportunities lie, but also where things can be improved”* (31-03-2021).

**Table 3:** Average participant evaluation

	Average evaluation on a scale from 1 to 5 (1 = fully agree, 5 = fully disagree)						
	The tool helped to form a collective ambition	The tool helped to learn about implications on the multi-stakeholder network	The tool helped to explore and negotiate matches and mismatches between stakeholders	The tool helped to define first interven-tions	The tool is easy to use	The boundary cards were useful to support the tool	The meaning of the different organizational boundaries were clear to me
Trial runs	1.9 (usefulness for purpose)				2.1	1.7	2.4
Internal runs	3.2	2.8	2.6	2.4	2.6	3.2	3.2
External runs	2.4, *σ* =1.14	2.3, *σ* =0.86	2.4, *σ* =1.13	2.3, *σ* =1.21	2.2, *σ* =1.11	1.9, *σ* =0.89	2.3, *σ* =0.95

The tool also triggered two different participants to share the tool with others within their organization. In one situation, the sharing was intended to enable follow-up on interventions: *“I am also going to bring this tool to the attention of colleagues to convert structured matches and mismatches into interventions”* (participant of Lightronics workshop, 29-03-2021). The other situation might point at a wider need for company employees to be assisted in connecting with external stakeholders. As the participant of the Philips workshop pointed out: *“Very nice way of structuring the workshop and align thoughts. Easy to use. I will introduce this within my organization”* (15-04-2021)*.*

While the online format provided an effective way to include multiple stakeholders, we felt that it made it more challenging to let the participants see the whole picture from the beginning and to make them familiar with the technical tools and working format. After each iteration, we adjusted the workshop and tool based on the quantitative feedback in Table 3, and the qualitative feedback in Table 4.

**Table 4:** Workshop feedback and development

	**Feedback**	**Adjustments made**
**Expert panel**	• Boundary of power and identity seems most challenging to change. Focus on in workshop. • Many insights on boundaries (content, framing, relevance) • Include triple bottom line identity • Identify changes needed on company and network level. Mark the crucial changes. • A joint purpose, shared identity and intrinsic motivation is important • Include social & technical competences • Think about strategic options: change/strengthen/disrupt • Include what one can do themselves and what they need others for • Create letter of intent	• Created tool and workshop • Adjusted ‘identity’ to ‘identity & mindset’ • Adjusted ‘power’ to ‘influence & control’ • Adjusted ‘competence’ to ‘materials & skills’ • Included multi-purpose in identity boundary card • Formulated facilitating questions for the different boundaries and activities • Included questions about own contribution vs. contribution of others • Created a collaboration pitch with own contribution in relation to others’ contribution • Added ‘matches & mismatches’ to the workshop
**Trial runs**	• Think about how to process the information in the central tool • Include implementation of proposed ideas • Pre-define goal of the workshop • Translate for Dutch participants • More elaborate description and examples of the boundary cards • Include break to digest before defining matches/mismatches • Nice way to stay connected in a digital pandemic world • Include test to work with the digital platform and if possible technical assistant	• Included the formulation of a common goal in which profit and purpose is balanced • Included first interventions after (mis)matches • Revised description of the boundary cards to enhance differentiation • Revised example • Included workshop purpose in instructions • Included a 10-min break time before defining matches/mismatches • Translated into Dutch • Included platform test at the start of the workshop
**Internal runs**	• More time to define the ambition • Enhance conceptual clarity of boundary cards • Prioritize (mis)matches • More discussion time needed (the workshop can easily be split into 3 times 2 h separate sessions)	• More elaborated description of the boundary cards to enhance conceptual clarity. Added a (digital) backside to the card with more examples. Positioned the cards next to the tool • Adjusted ‘influence & control’ to ‘relations & contracting’ • Changed sequence of boundary mapping from identity towards efficiency • Included voting to focus (mis)matches • Extended time for negotiation to 30 min
**External runs**	• Explain the focal stakeholder network as each actor in the network has its own stakeholder network too • Spend a little more time on training	• Added the related stakeholder network to the workshop purpose in instructions • Avoid too abstract ambition in pre-workshop conversation • 2.5–3 h for the workshop • For the future: create webinar to prepare the workshop

### Impact of the Tool

In the past, rigorous testing and practical validation of tools in business innovation were not self-evident [20, 38]. Recent developed tools for SBMI were tested in practice and evaluated based on whether the users found the tools useful and easy to use [17, ]. This study advances the testing of the tool by integrating the *outcomes* of the tool intervention, building on the importance of reflection and learning on relationships between the company and its external environment to integrate these insights into business decisions [38, 59, 65]. Therefore, we inquired the impact on the users directly after the workshop and 1 week afterwards. Instead of measuring the impact of the tool in a quantitative way (e.g. number of interventions defined), we asked what participants had learned, realized and decided, and what actions they had taken after the workshop. Illustrative quotes from the learnings indicated by the participants directly after the workshop are presented in Table 5.

**Table 5:** Illustrative participants’ learnings directly after the workshop. The feedback process itself was anonymous

*What have you learned from applying the tool?*	
**Trial runs**	• Mainly, I believe this tool is useful to gain insight in who the most valuable potential stakeholder are, and what needs to be done with them. For example, in our case the waste-processer proved to play a vital part in any solution, while the contribution of citizens was marginal in the first stage. This helps in identifying where most attention can be spent. • That all the different stakeholders have different aims and goals, and that it is tough (but possible) to create collective goals that will benefit all the stakeholders involved. Also, it will be useful for future plans, to be able to look back on the work that has been done in previous workshops/etc. • Confirmation of the necessity and strength of the stakeholder approach, especially in the circular transition. • The tool readily showed the different organizations and their interlinkages, as well as the need for a systemic view for any project (especially circular). • I realize better now that all stakeholders look from their own perspective. Despite the common purpose, the direction of ideas and thought is very different from everybody’s perspective. • I learned a lot about organizational boundaries and that change is needed to apply new, circular business ideas. The tool helps to formulate those changes. • I learned that collaboration between stakeholder relies heavily on understanding their perspective boundary, and control over resources and assets.
**Internal runs**	• We are well underway but often do things based on experience but not based on knowingly applying them as a tool. • I found it very educational to work from efficiency to identity. • Make a clearer distinction between certain ideas and where they belong in the organization. • Formulate or find out concrete problems/solutions. • Converting a general ambition into concrete interventions
**External runs**	• Deeper insights in potential to leverage other organizations’ strengths and opportunities very nice way of structuring the workshop and align thoughts • That it is an immense challenge to improve a system with such a broad range of stakeholders and problems. We went from software to shipping and from local use to Dutch regulations—let alone discuss the hard-core financials. • Find opportunities and difficulties in the boundaries. • Where we looked at how we can tackle these together through consultation with various parties, it suddenly becomes clear where opportunities lie, but also where things can be improved. • That by exploring this in this way you can get to know each other and cooperation opportunities. • To provide insight into opportunities and threats and to link them to new action points

Following up shortly after the external workshops, we inquired the initiating companies with the following questions:

- What realizations have come to you in the past week?
- Have there been internal or external follow-up meetings?
- Have choices been made? (e.g. to continue, to focus, to go one step further)
- Is there something else you want to share?

The short-term impact assessment showed that the tool made participants realize which visible and invisible elements their organization had to strengthen and/or let go. For example, Arveco expressed that *“We realize that we need a new brand”* and that the workshop made them *“choose a path, what do I have to let go, what falls into place”* (Arveco representative, personal communication, 31-03-2021). Additionally, the tool helped to gain confidence to start new partnerships, as was illustrated by Philips *“It is a complex issue but there is willingness in the network to address and cooperate”* (Philips representative, personal communication, 19-04-2021). Also, City Farm Brandvoort expressed this function of the tool *“The* w*orkshop has given a boost in confidence for cooperation in the future”* (City Farm Brandevoort representative 1, personal communication, 13-04-2021) and *“I realize, hey, there is really energy in these people, they really want to. But the parties also learn from each other that this energy is in it, so not just Brandevoort as a connecting factor”* (City Farm Brandevoort representative 2, personal communication, 13-04-2021)*.* A representative from an external stakeholder of Lightronics expressed *“I hope we get to proceed on the generated ideas and am curious whether and how it will be followed-up”* (Hydro representative, personal communication, 25-03-2021), while the representative from Lightronics stated that they will have to await the students’ research results to get approval from the management but also stated that *“there is again talk of light as a service”* (personal communication, 06-04-2021). Except for Lightronics, all cases held follow-up meetings with potential partners within 7 days after the workshop. Philips and Arveco established a new working group as self-managing multi-stakeholder teams to work on the interventions: *“We organize an open invitation to tune-in with each other after two months”* (Philips representative, personal communication, 19-04-2021). The same representative also expressed that it is important to find and connect to the right people within the different organizations to organize such follow-up.

## Discussion

This study presents a boundary tool that contributes to SBMI research and practice. The tool bridges the theory and practice by integrating exploratory, negotiation and agreement activities on the four organizational boundary dimensions in relation to a collective SBMI ambition.

The theoretical contribution of the tool lies in the integration of a multi-stakeholder boundary work perspective to existing SBMI approaches and its translation into entrepreneurial linguistics. The tool has the potential to integrate both values-based network and business model innovation approaches [28] as well as effectuation approaches [17, 66], depending on the sequence of boundary mapping. Additionally, this study contributes to tool development for SBMI by taking an explicit (multi-)stakeholder focus and integrating its visible (e.g., materials, costs, machinery) and invisible (e.g. mindset, power and trust) organizational boundary phenomena. This makes the tool well suited as a follow-up exercise for value proposition and strategy-based SBMI tools, such as the recently developed Circular Card Desk [63], which excludes social and institutional actors, the value mapping tool [32] or the collaborative circular proposition tool [17]. The boundary tool can be proceeded by experimentations on the desirability, feasibility, viability and sustainability of the configuration [43]. Additionally, this study advances research on SBMI tool development by proposing a research strategy to inquire the impact of tools, called for in previous research [20]. The output of the tool is not a clear-cut process outline but leads to critical realizations and follow-up actions for the purpose of SBMI. The visualizations and interventions defined in the tool may function as artefacts to further design and assess the organizational boundary changes in the multi-stakeholder network in a collaborative process, keeping the SBMI ‘alive’ [67, 68].

The practical contribution lies in presenting a well-tested boundary tool that assists companies in engaging with multiple stakeholders for the purpose of SBMI. The tool enables interactive, collaborative exploration and stimulates the thought-process and the development of narratives in preparation for (bottom-up) actions [69]. Through the boundary work tool, visible and invisible aspects can be explored and addressed. Based on our workshop observations, we can infer that the tool helps companies to become more aware of their own boundaries in relation to their stakeholder environment, and to respond to it more strategically. For example, by making decisions on the organizational purpose, by including end-of-life contracting and the redistribution of roles and activities through partnerships. Additionally, the tool stimulated joint discussions on stakeholder’s individual challenges for alignment, leading to a shared commitment for finding solutions. This was illustrated by a light provider, which struggled to grasp the implications of reversed logistics of street fixtures on their competences and activities, and to find a way to implement these changes. In response, another participant offered their expertise and proposed to discuss the sharing of their own reversed logistics operations as well as the exchange of returned materials, something that would otherwise not have been discussed. A field of tension is whether it feels safe enough for the initiating organization to open up the relationship to other stakeholders and bring multiple value chain partners together in one workshop. The case of Lightronics showed a tension from a potential supplier to invite municipalities (as procurers). This is where the role of the facilitator was challenged towards politics, diverting from a broker towards becoming an issue advocate [70].

While the purpose of the tool is to help companies engage in or intensify multi-stakeholder collaborations for sustainable business model innovation, the internal runs showed that the tool can also be used to find business model opportunities within the company’s own boundaries. In addition to companies aiming for strategic innovation, potential users are intermediaries in the sustainability field, trying to promote sustainability with sense making, conveying the message of collective action and collaboration, and change agents aiming for interventions in mission-oriented innovation systems [71].

The tool is flexible to engage different types and numbers of stakeholders in the workshop, although for large or multiple stakeholder networks adjustments and reiterations are needed to facilitate broad-based participation. However, the tool also has its limitations. First, boundary work for SBMI is a long and reflexive process that can easily take multiple years to lead to alignment of the critical stakeholders. While a single tool cannot easily organize such a complex process, the boundary tool provides for a comprehensive starting point of multi-stakeholder collaboration and can be used to assess the progress on stakeholder alignment during the SBMI process [59]. Second, the workshop should be facilitated by someone who is familiar with the organizational boundary framework. This relates to the third limitation: organizational boundaries emerge in different forms depending on the various contexts and SBMI ambitions. Currently, there is no complete ‘indicator list’ per boundary or typical boundary configurations per type of actor.

Further research could enhance organizational boundary thinking by inquiring the typical boundary indicators and boundary reconfigurations in specific contexts and for specific actors, advancing recent research [23–25, 72]. This research would enable further customization per strategic application [66] and per type of boundary work, such as identity work [46], or power work [39, 47, 61], but also enable a more independent use of the boundary tool by practitioners. A potential theoretical avenue that helps to navigate tensions in SBMI is the field of paradox theory, which focuses on the accommodation of interrelated and conflicting economic, environmental and social concerns involved in achieving business contributions to sustainable development [73–75]. Building on recent work that integrates paradox theory into SBMI and circular business models [75, 76], an interesting research avenue lies at expanding paradox theory from managing paradoxes at the organizational level towards managing paradoxes at the inter-organizational level, encountered by multiple stakeholders simultaneously, which is the domain of boundary work. For example, paradox theory could help resolve boundary issues between multiple stakeholders in SBMI and the principles and building blocks of boundary work could be used for future research on tools and theory development [75, 76]. Additionally, further research could integrate the boundary tool in experimentation with circular service-driven business models to enable iteration on multi-stakeholder value propositions [39, 77].

## Conclusion

Multi-stakeholder alignment is imperative for capturing the sustainability potential of SBMI, but few studies offer guidance to companies for engaging in such a collaborative process. Organizational boundary work provides a novel perspective to explore, negotiate and align stakeholders for this purpose. This study presents a tested boundary tool that helps companies to engage in and/or intensify multi-stakeholder collaborations for SBMI. For companies, key benefits of the boundary tool are: (1) a re-design of the multi-stakeholder system based on a collective SBMI ambition, (2) an assessment of a companies’ own organizational boundaries and its multi-stakeholder network, (3) the exploration, negotiation and prioritization of strategic actions based on organizational boundary changes, (4) the exploration of new partnerships and (5) a kick-start to engage with these partners. Engaged scholarship into multiple contexts is needed to further improve the boundary work approach and the boundary tool.

## Figures and Tables

**Fig. 5:**
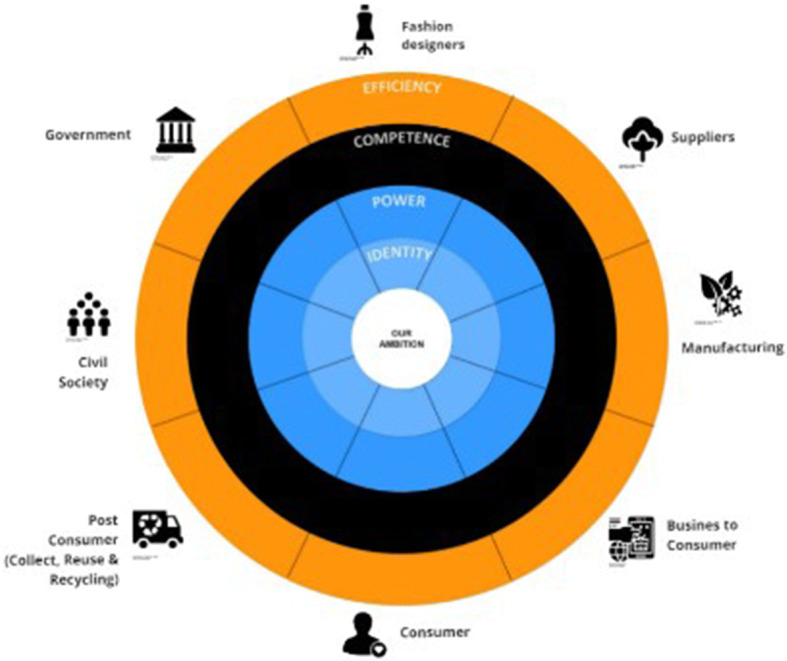
Boundary Work Tool

**Fig. 6:**
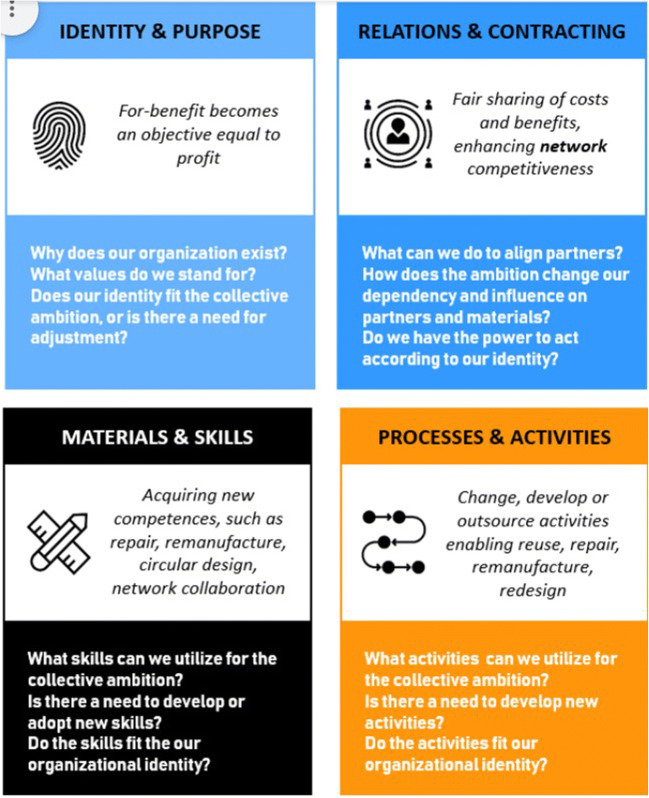
Organizational boundary cards

**Fig. 7:**
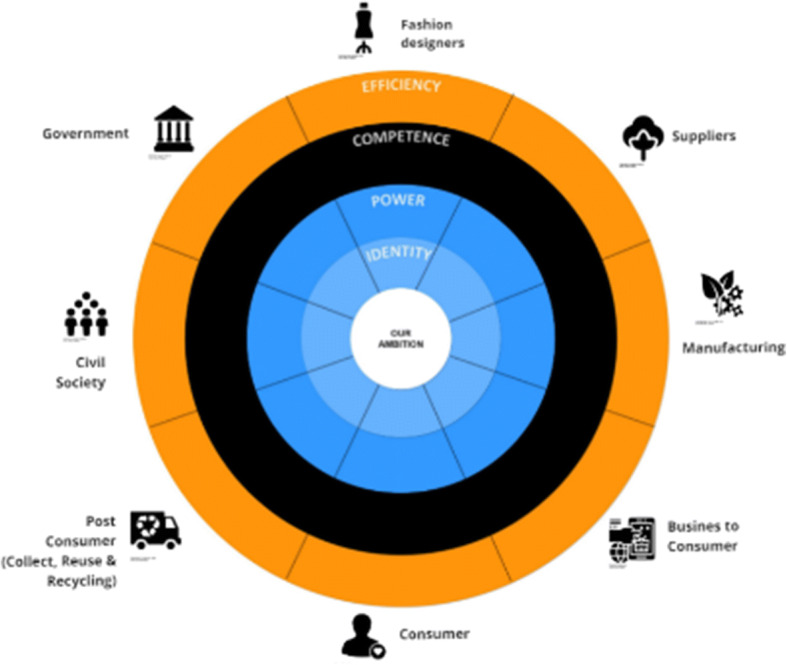
Boundary Work Tool

## Data Availability

Not applicable.

## Appendix 1. Digital tool assessment form initial tool design

### Tool Assessment Form

You have just used the boundary work tool. The purpose of the tool (Figure 5) is to learn about circular opportunities in the multi-stakeholder network based on the changing organizational boundaries, and to explore boundary matches and mismatches between stakeholders. The tool is supported by organizational boundary cards (Figure 6).

1. The boundary work tool is useful to address the purpose stated above:
  - *Fully agree ○ ○ ○ ○ ○ fully disagree*

Please explain your answer (What was most useful? what was less useful? why?)

2. The boundary work tool is easy to use:
  - *Fully agree ○ ○ ○ ○ ○ fully disagree*

Please explain your answer:

3. The boundary cards were easy to use
  - *Fully agree ○ ○ ○ ○ ○ fully disagree*

4. The meaning of the different organizational boundaries were clear to me
  - *Fully agree ○ ○ ○ ○ ○ fully disagree*

5. What have you learned from applying the tool?

6. Do you have any recommendations for workshop and tool improvement? What should be started/stopped/considered or continued?

## Appendix 2. Digital tool assessment form improved tool design

### Tool Assessment Form

You have just followed the workshop ‘boundary work’. The aim of the workshop is to formulate a joint ambition and to explore and negotiate the needed changes, opportunities and tensions in the multi-stakeholder network, as a basis for finding first interventions.

To improve the tool (Figure 7) and workshop, we would like to ask you to fill in the questions below.

1. **The workshop helped to form a collective ambition**

*Fully agree ○ ○ ○ ○ ○ fully disagree*

Please explain your answer:

2. **The tool helped to learn about necessary changes in the multi-stakeholder network**
  *Fully agree ○ ○ ○ ○ ○ fully disagree*

Please explain your answer (What did the tool do well/what could be improved?)

3. **The tool helped to explore and negotiate opportunities and tensions between stakeholders**
  *Fully agree ○ ○ ○ ○ ○ fully disagree*

Please explain your answer:

4. **The workshop helped to define first interventions**
  *Fully agree ○ ○ ○ ○ ○ fully disagree*

5. **The tool is easy to use:**
  *Fully agree ○ ○ ○ ○ ○ fully disagree*
6. **The tool is supported by organizational boundary cards (see figure below). To what extent did you find these cards useful to support the tool?**
  *Very useful ○ ○ ○ ○ ○ not useful at all*
7. **The meaning of the different organizational boundaries were clear to me**
  *Fully agree ○ ○ ○ ○ ○ fully disagree*

**8. What have you learned from applying the tool?**

**9. How can the workshop and tool be improved? What went well, and what should be started/stopped/or adjusted?**

## Appendix 5. Guiding lines and questions for the workshop

**Table:**

Phase No Step		Guiding lines and questions
Exploring	1. Set the scene and get started	Why are we here? What are we going to do? In five steps we will create a shaper perspective on our collective ambition, and explore how we can contribute to this ambition and where we need to jointly change aspects of what we do. Which stakeholders are relevant for this ambition? Go all the way downstream and upstream, and don’t forget societal stakeholders such as public organizations, environment organizations, citizens, consumers. Are there stakeholders that become redundant? Whom at the table represents which stakeholder?
	2. Develop a collective ambition	How would we define the sustainability ambition of this group? What are modest improvements that we can already do for this ambition? What do we want to go into further that requires significant changes?
	3. Explore boundary changes	How does the ambition affect your organization boundaries? Sketch how your future organization look like using the OB cards. Keep in mind that your future boundaries should contribute to the collective purpose. You can build on your existing boundaries, but probably these will need to be adjusted more or less. For example, are you still a producer of products, or rather a circular concepts developer? Write your future boundaries down with post-its and map them on the boundary tool. Lets’s start with the boundary of identity... How do you the identity of your organization in this future? Does your current corporate identity and mindset fit the ambition? If so, how? If not, how should our purpose be changed to improve alignment? How does the innovation change your organization’s influence on stakeholders and/or material? Whom are you steering and whom do you depend on? Does your organization have the influence to align others? What can your organization do in terms of contracting? How can you as a network enhance your competitiveness? What competencies, skills and materials does the innovation require form your organization? Are there new materials or skills to be developed? Do you aim to develop these yourselve, or can others help with that? Does this still fit within your organizational identity? What (novel) activities could your organization deploy for this ambition? What do you do differently in five years? What are the implications for the organization’s processes? Do you deploy these activities yourself or are other needed? Do the envisioned activities still fit your envisioned organizational identity? Name three things that you notice. Can you share what you can do yourself, and what you need others for?
Negotiating	4. Negotiate boundary changes and identify matches and mismatches	Where do you see the greatest possibilities? Where do we see opportunities to get to get started easily? What takes more time? Where do we see obstacles? Are they crucial? Who feels its difficult to align? What still needs to be found out? Who’s purpose seems contradictory with the collective purpose? What mission or vision statement is challenging here? How is power being shared amongst us? whom has control/influence over others? Whom should be empowered and whom can help with that? Whom/where should power be declined? Whom has the power to align others? How do we share information? Who has skills, networks or machinery that could be useful for another organization? Are there stakeholders from other markets who could have relevant competences? Will we need a new type of stakeholder? How does this change the activities of stakeholders in our network? Could suppliers adopt a repair/remanufacturing role? Who will be responsible for reversed logistics? What different consuming habits have to be nudged for?
	5. Prioritize matches and mismatches	In the previous discussion, we have identified the following matches and mismatches. Do we still have any additions? Which matches would you like to get started with right away or have major potential? What else do we have to do or find out? Which mismatches are critical?
Implementing	6. Define first interventions	Match: For the topic of X, what could be way to start with capturing this opportunity? Mismatch: For the topic Y, what can we, or others, do to address this tension? Do we need to align our collective purpose?
	7. Create a collaboration pitch	We can now fill the collaboration pitches for the different interventions.
